# Nurses’ views and experiences of caring for malnourished patients in surgical settings in Saudi Arabia – a qualitative study

**DOI:** 10.1186/1472-6955-13-29

**Published:** 2014-10-13

**Authors:** Atika Khalaf, Albert Westergren, Örjan Ekblom, Hazzaa M Al-Hazzaa, Vanja Berggren

**Affiliations:** 1 The PRO-CARE Group, School of Health and Society, Kristianstad University, Elmetorpsvägen 15, S-291 88 Kristianstad, Sweden; 2 The Department of Public Health Sciences, Karolinska Institute, Stockholm, Sweden; 3 Åstrand Laboratory of Work Physiology, The Swedish School of Sport and Health Sciences, Stockholm, Sweden; 4 Paediatric Exercise Physiology Research Laboratory, College of Education and Obesity Research Chair, King Saud University, Riyadh, Saudi Arabia; 5 Department of Health Sciences, Medical Faculty, Lund University, Lund, Sweden

**Keywords:** Malnutrition, Physical activity, Saudi Arabia, Nursing, Nurse’s views

## Abstract

**Background:**

Although the occurrence of malnutrition in hospitals is a growing concern, little is known about how hospital staff understand the care that nurses provide to patients with malnutrition. The purpose of this study was to explore nurses’ views and experiences of caring for malnourished patients in Saudi Arabia (KSA).

**Methods:**

Using a qualitative explorative design, fifteen nurses were interviewed as part of a purposive sample hospital staff. The transcripts were analyzed using latent content analysis.

**Results:**

The nurses spontaneously and consistently linked malnutrition with physical inactivity. The two main categories, which emerged, were: ‘Potentials for nurses to provide good nutrition and physical activity’, and ‘Having the ability but not the power to promote proper nutrition and physical activity’. These arose from the subcategories: Good nursing implies providing appropriate health education; Acknowledging the *Mourafiq* (sitter) as a potential resource for the nursing, but also as a burden; Inadequate control and lack of influence; Cultural diversity and lack of dialog; and Views of women’s weight gain in KSA society.

**Conclusions:**

The nurses felt they have the capacity and passion to further improve the nutrition and activity of their patients, but obstacles in the health care system are impeding these ambitions. The implications for nursing practice could be acknowledgement of the nurses’ views in the clinical practice; culturally adjusted care, improved communication and enhanced language skills.

## Background

In high-income countries, concern is growing about the occurrence of malnutrition in hospitals [1,2]. Simultaneously, there seems to be little knowledge about how hospital staff reflect on nursing care provided to patients with malnutrition [3]. Malnutrition can be defined as insufficient, excessive or imbalanced consumption of nutrients [4]. Thus, malnutrition does not only include undernutrition, but also overweight/obesity and nutrient deficiencies.

In health care today, malnutrition of hospitalized patients is a widespread problem that results in serious or adverse health outcomes [5,6]. Malnutrition has been documented in hospitalized patients over the last two decades [7,8] and is associated with an increased prevalence of complications and high mortality [9]. However, precise information about the frequency and severity of malnutrition in hospital patients is difficult to obtain [9-11] despite growing awareness that a patient’s nutritional status may worsen during a hospital stay [9-11]. In the previously referenced studies [9-11] hospitalized patients at risk of undernutrition did not get expected nutritional care, i.e. oral supplements, energy-enriched meals etc. Thus, the role of nursing has become a central concern. Nurses typically assist patients with food intake by screening patients’ nutritional status at admission; providing an individualized nutritional care plan; assessing each patient’s ability to eat upon admission; ensuring that there is a focus on the patient’s mealtime; providing adequate nursing or other support in assisting at mealtime; encouraging and assisting patients to maintain their oral care as well as managing mealtime environments [12]. Despite these research findings, little is known about the nursing care provided for malnourished in-hospital patients in the Kingdom of Saudi Arabia (KSA).

The provision of good nutrition care by nurses has been reported in different studies. A study in the United States involving nurses and nursing assistants assessed the perceived beliefs and views related to patients’ nutritional needs and found that specific barriers such as lack of time and training, working short-staffed, poor food quality, and a lack of nurse to nursing-assistant teamwork may affect the nutritional routines provided by health care staff [13]. A Swedish study showed that undernutrition as such was seen as a “taboo” – a finding that possibly contributes to decreasing the likelihood of proper identification of undernutrition and provision of good nutritional care [3]. This indicates the importance of further highlighting nursing staff’s experiences of the care provided to malnourished hospitalized patients. In health care institutions, the nurses’ knowledge and attitudes as well as routines are likely to have an impact on the nutritional care provided [14].

The concerns about the occurrence of malnutrition in hospitals have been confirmed by Australian researchers in a study where they hypothesized that reduced involvement of nurses in patients’ nutritional care may be one of the factors contributing to the development of malnutrition among hospitalized patients [1]. The study revealed that many nurses lacked the in-depth knowledge needed to provide proper nutritional care to their patients. They also found that, although nurses considered nutritional care to be important, many had difficulty in raising its priority above other nursing activities, as a result of time constraints and multitasking issues [1].

Furthermore, when discussing KSA health care system we find that more than 80% of the physicians and nurses who are employed by health services in KSA are expatriates [15]. These health care staffs from diverse cultures bring a variety of experiences to the practice settings [16,17]. Since KSA as the rest of the world becomes globally multicultural, transcultural nursing that is directed toward holistic and congruent health care is challenging nurses and other care givers to think broadly so that ethical and moral factors become clearly evident as one works with patients from different cultures [18]. Health care professions need therefore knowledge of the complex social structure, world view and cultural context of the people of KSA in order to promote culturally congruent care [17,19]. Building on these facts one can argue that there is a need to raise nurses’ awareness of the importance of transcultural nursing also when caring for the patients’ nutrition to promote health, healing and well-being.

A literature search conducted in November, 2013 using relevant scientific databases (PubMed, Cinahl, ScienceDirect, and Google Scholar) on the topics of “nutrition and malnutrition” in relation to “nursing, caring, and nursing care” only revealed three studies [7,8,20] explicitly concerned with patient malnutrition in Saudi Arabia. These studies reported on the circumstance of adolescent girls in eastern KSA and males living in the Riyadh Nursing Home. Because of the lack of qualitative studies about the nutritional care of in-hospital patients in KSA it is not surprising that little is known about nurses’ views and experiences of caring for malnourished patients in surgical health care settings in KSA. The aim of this study is to explore nurses’ views and experiences of caring for malnourished patients in health care settings in KSA.

## Methods

This study explored views and experiences of caring for malnourished patients in surgical health care settings in southwestern KSA. In order to capture the nurses’ experiences, an inductive qualitative approach with an interview-based procedure was chosen [21].

### Data collection

#### Sample

The selection of nurse participants was made in collaboration with a nurse supervisor working in the Nursing Education Department according to purposeful sampling principles [22]. The purposive sampling focuses on particular knowledge and experiences of individuals that are of interest, i.e. the individuals who are most informative in relation to the research question [22]. After the first four pilot interviews, a snow ball technique was used to recruit the informants [22] where the interviewed nurses asked colleagues to participate in the study. The interested nurses were then given written information letter including a short background on the topic, the aims and relevance of the study, and the definition of malnutrition used in current study. Prior to the interviews the participants were also given the opportunity to ask about the given information. We included a variety of participants as regards sex, age, nationality, total job experience as nurses in years, job experience at current ward (Table 1).

**Table 1:** Characteristics of participants

**Participants**	**n = 15**
**Sex**	
Women	12
Men	3
**Age**	
Median (min–max)	30 (24–53)
**Total job experience**	
< 1 year	1
1 – 5 years	8
5 – 10 years	2
> 10 years	4
Median (min–max)	4 years (6 months–26 years)
**Job experience at current ward**	
6 months – 5 years	13
6 years – > 10 years	2
**Wards**	
Female general surgical ward	5
Male general surgical ward	3
Male orthopedic ward	7

#### Study context

KSA is a country that has seen rapid development in the last few decades in the areas of industry, education, and healthcare services [23]. However, KSA lacks well-trained native KSA healthcare providers [24] that is similar to the severe shortage of nurses in most developing countries [16]. This shortage has led KSA to rely on expatriate nursing staff [16] as a way to overcome the shortage of native nurses [25], and to cope with the dramatic changes facing KSA as a result of industrialization and urbanization [16].

This study was carried out in the present analysis is the biggest governmental hospital (about 658 beds) in southwestern Saudi Arabia. As a referal and tertiary hospital, it is about 25 years old, has many subspecialties and provides 24-hour emergency care. The official language used for communication and documentation in health facilities throughout KSA is English [15]. When patients are admitted, especially females, they are usually accompanied by a sitter, who is most often the patient’s relative. The companion, (sitter), assists the patient in his or her daily routines and is called a *Mourafiq* in case of male patient or a *Mourafiqah* in case of female patient [15]. Furthermore, nurses, whether local or expatriate, are usually educated in English.

#### Procedure

The study was conducted in two phases. The first phase was a pilot phase that included four semi-structured interviews. These were held in order to examine and develop the thematic issues that were the focus of subsequent interviews [22]. Two of the pilot interviews were transcribed directly, which led to a slight modification of the interview guide. These were namely the deletion of two questions that were identified as difficult to understand by the participants or irrelevant. The questions asked covered four themes; 1) knowledge and reflection of malnutrition in health care, 2) the experiences of practical nursing care in relation to surgical patients’ nutrition, 3) reflections on and experiences of ethical problems in caring for surgical patients’ nutrition, and 4) professional challenges in the context.

The pilot interviews phase was followed by eight semi-structured interviews. Finally, a further three interviews were completed in order to reach saturation, i.e. when no further variation or any additions to the statements were found by the research team [22]. All the interviews took place, after agreement with the participant nurses, on the ward in a conversation room and were tape-recorded. The interviews were conducted by the first author in English or in Arabic depending on the wishes of the respondent. A total of eleven interviews were conducted in English and the other four in Arabic. The time for each of the interviews varied between 42 minutes and 68 minutes. All interviews took place during the period January 5 to June 17, 2010. In total fifteen interviews were held and all were included in the analysis.

### Analysis

Latent content analysis was chosen as the method to reveal deeper or latent meanings in the text [26]. Both manifest and latent content analysis include an interpretation of the text, although both vary when it comes to the depth and level of abstraction of the interpretation [27]. Moreover, qualitative data analysis tools allow for a more rapid and rigorous qualitative data analysis [28]. For assistance in the analysis we used Atlas-ti as an initial sorting assistant program because of its essential capabilities for different types of analysis [29].

The texts in English were first read through by three of the authors individually in order to get an understanding of the whole. On the basis of the first naive reading, certain thoughts and reflections were distinguished in the texts. These first thoughts and reflections were recorded in the margins and were used continuously during the remaining stages of the analysis process. The material was discussed and analyzed stepwise with the aim of the study in mind. Meaning units were then identified [27], i.e. assertions from the content of the text. Meaning units with similar content were organized into different issues with the study’s aim in focus. Comparisons were made to find similarities and differences in order to get a deeper understanding of the underlying text in the latent analysis. The meaning units were condensed (the sentences were shortened while the core was preserved) [26]. Analyzed material was sorted into subcategories. In the last stage the subcategories were linked together in two main categories that expressed the latent content. After time and rereading, a pattern was seen embedded in all the findings, recognized as the overall understanding.

### Ethics

The Helsinki Declaration’s [30] ethical recommendations were followed during the planning and implementation of this study. This study was approved by the Hospital’s local ethical committee (No. 50/80/26889), and by the Ministry of Health, KSA (April 20, 2009). The nurses participating in the study were asked for and signed an informed consent. Both verbal and written information was given about the voluntary participation and the nurses’ right to withdraw at any time during the study without giving any explanation. The nurses were also guaranteed confidentiality, i.e. no personal identification number or names were collected.

## Results

### Characteristics of the participants

All nurse participants in the study were registered clinical nurses of different nationalities; the Philippines (n = 10), KSA (n = 4) and from India (n = 1). Table 1 shows the characteristics of the nurses interviewed for this study.

### Main findings

Two main themes emerged from the study: 1) Potentials for nurses to provide good nutrition and physical activity, and 2) Having the ability but not the power to provide good nutrition and physical activity for patients. These main categories embrace five sub-categories (Table 2). Although it was not the aim to explore the nurses’ views and experiences of the patients’ physical activity, it happened recurrently that the nurses spontaneously linked malnutrition with physical activity and inactivity. From the nurses’ perspectives malnutrition and physical inactivity were interconnected and the overall understanding from the analysis was *Nurses’ potentials and barriers to provide proper nutritional care*. The undernourished patients were thought to be less physically active because they lacked the energy to expend on physical activity. On the other hand, nurses thought the overweight patients to be less active because of different priorities. These patients for example were thought to prefer sitting and chatting to being physically active. The nurses’ views of physical activity are therefore described along with their nutrition-related experiences.

**Table 2:** The nursing staff’s experiences of caring for malnourished patients at surgical settings in a governmental Saudi Arabian hospital

***Nurses’ potentials and barriers to provide proper nutritional care***	
**Main categories**	**Subcategories**
**Potentials for nurses to provide good nutrition and physical activity**	*Good nursing implies providing appropriate health education*
	*Acknowledging the* Mourafiq *as a potential resource for the nursing, but also as a burden*
**Having the ability but not the power to provide good nutrition and physical activity**	*Inadequate control and lack of influence*
	*Cultural diversity and lack of dialog*
	*Views of women’s weight gain in KSA society*

### Potentials for nurses to provide good nutrition and physical activity

Potentials according to the nurses consisted of acknowledging the relatives as a resource in the practical nutritional care provided. Perceiving health education as a key tool for nutritional problems and physical inactivity was also identified as a potential for the nurses to provide good nutrition and physical activity.

#### Good nursing implies providing appropriate health education

Nurses identified their role in the organization, building on their responsibilities. The expression “being a nurse” was spontaneously explained many times during the interviews in that nurses viewed their important presence for the patients and for the organization as a whole. The nurses perceived their nursing role as providing good care and expressed that in how a nurse was expected to be or to act. To provide good nursing care, the interviewees emphasized that the nurse should know the reasons for and possible diagnosis of undernutrition as well as overnutrition to be able to provide health education to patients including advice about a healthy and active lifestyle. This knowledge gave the knowledgeable nurse a powerful role in decision making and in patient compliance, according to participants. Further, almost all participants stated that both patients and their relatives would need continued and repeated health education, especially about nutritional issues.

Moreover, another repeated story told by nurses was that nurses should be living examples of a healthy lifestyle. In addition to their personal choice of lifestyle, a nurse should preferably use all possible opportunities to educate patients and encourage them to improve their lifestyles with respect to nutrition and physical activity.

*“Those who are underweight are usually weak and need health education. We can tell the patient about the best types and amounts of food that she should eat. And if the patient has problems with being overweight, then she can also benefit from health education”* (Interview 8)

*“At the same time, we encourage them to be more physically active … because we are there to guide and support them”* (Interview 6)

### *Acknowledging the* Mourafiq *as a potential resource for the nursing, but also as a burden*

The nurses expressed mixed feelings about the role of the *Mourafiq*. However, nurses experienced having a sitter/relative present around the patient all the time as mostly positive. This is because the sitter offers support and care to the patient. As one nurse put it, “*they give a lot of care*” (Interview 7).

The hospital in which the study was carried out did not have a dietician. Nurses frequently mentioned the absence of this key role in patient care along with the shortage of nurses as significant. These circumstances meant that the role of relatives in caring for patients’ nutrition became so central that many of the nurses interviewed considered relatives to be a resource to the nursing team. Such nutritional care found expression in feeding, bringing food from outside hospital the patient might desire and by advocating on behalf of the patient with respect to food choice, hot food etc.

Likewise, a patient’s *Mourafiq* was the one who could bring a patient alternative food. Nurses held different impressions about food from outside of the hospital. Some nurses considered it tastier than available hospital food, thus helping patients avoid undernutrition. Other nurses thought that food from outside of the hospital must be controlled so that patients would not develop overnourishment or other health related complications.

*“Here it’s always the relatives who influence the patients’ diets because they are always bringing in food from outside of the hospital”* (Interview 9)

### Having the ability but not the power to provide good nutrition and physical activity

Nurses described several barriers to their providing proper nutritional care and physical activity to hospitalized patients. Nurses identified the expected (typical) social role of women in KSA to be one of the obstacles female patients faced, especially with respect to leading a healthy lifestyle, which would include taking exercise and eating properly. Another barrier nurses described was their perception of not having adequate control over their patients’ food intake. Further, nurses described feelings of lack of dialog between themselves and patients, relatives, colleagues, and other healthcare professions to be culture- and language-based.

#### Inadequate control and lack of influence

The nurses described many situations in which they experienced inadequate control over patients’ food intake. One such circumstance arose a result of relatives’ involvement in patients’ nutritional care. For example, relatives/sitters would bring fatty food for patients with chronic diseases such as hypertension and diabetes. Sometimes, nurses would view the *Mourafiq* as an impediment to the healthcare providers’ attempts to control food intake. Significantly, nurses denied hospitalization as having a role in patients’ developing malnutrition. Instead, they blamed the patients’ poor nutritional condition on the *mourafiqs’* involvement in feeding.

*“For example, if a patient is diabetic and is prescribed a fat-free diet but then the patient’s relatives give them meat and everything else high in fat, we are powerless to prevent the patient from consuming such an unhealthy diet”* (Interview 5)

Situations in which the nurses felt a lack of influence over patients’ food intake were sometimes related to organizational issues. The expectation of nurses to strictly follow orders given by medical doctors led to nurses feeling that they lacked adequate control over their basic responsibilities for patients’ food intake. Participants talked about being too dependent on medical doctors’ decision regarding nutritional issues. The nurses perceived themselves as only authorized to report the nutritional status of the patient to the doctor and as lacking the influence or mandate to consult a dietitian or a nutritionist to ask for advice and help. The nurse then had to follow physician orders that most often were not influenced by nurses’ reports. Nurses described situations where they almost gave up their responsibilities for nutritional care.

*“Then we discuss courses that the hospital could provide to update our knowledge, but what’s the use when the doctors’ orders have to be followed at the end of the day?”* (Interview 1)

#### Cultural diversity and lack of dialog

In the nurses’ narratives, cultural differences represent real barriers that affect and steal time from the provision of nursing care. These barriers were seen as related to differences between the nurses’ own cultures and the cultures of patients and other staff. Study participants also identified barriers related to language difficulties.

Nurses’ personal experiences of nutritional issues affected the care they would provide to malnourished patients. Having survived starvation, worked abroad and/or experiences of the cultural differences between Saudi Arabia and their own country of origin all emerged from our interviews as significant. Interviewees identified the separation of males and females as a cultural difference with clinical implications. Nurses reported the need to accompany a male physician encountering a female patient as time-consuming. The nurses also felt that patients would prioritize sitting and chatting over being mobile, which affected their nutrition negatively. According to the expatriate nurses, this sedentary behavior was probably due to cultural norms.

“*Overnourished patients, not only undernourished, they are not really active… you can see them sitting or even chatting with other patients”* (Interview 14)

Furthermore, nurses perceived their dialog with physicians and other healthcare staff such as nurse colleagues as inadequate. However, nurses felt communication with their patients to be less challenging than with their superiors. On the other hand, collaboration between nurses and other healthcare staff was something they desired more of in their jobs, although it did not always work as the participants wished. They ascribed this as a problem of cooperation and communication. To ease communication with the patients, nurses tried to use as many Arabic words they knew, but admitted that mutual understanding was limited because expatriate nurses lacked knowledge of the local Arabic language.

*“My work is also affected by the manner and style of communication among nurses and doctors. It is also affected when the patient won’t cooperate and when there are differences in culture or difficulties in understanding each other”* (Interview 2)

#### Views of women’s weight gain in KSA society

Both physical inactivity and increased food intake were held responsible for weight gain among Saudi Arabian women, according to the interviewed nurses. Nurses gave examples and stories about the expected role of women in KSA society. Women, according to nurses’ understandings of local cultural norms, bear life during pregnancy and so should be aware of proper nutrition to enable them to pass the knowledge of proper nutrition to her offspring. Culturally, women were described as expected ‘to eat for two’ during pregnancy, and that was stated as a reason why they would usually begin a negative, long-lasting path to weight gain. Some nurses indicated that women usually gain weight after marriage, while other nurse participants talked about KSA social expectations that women restrict their movement and that they would increase their food intake during leisure time.

*“Pregnant women also tend to become overweight because the women believe that if they eat more they will nourish the baby”* (Interview 12)

*“There are some limitations here… they have limited movement”* (Interview 10)

## Discussion

For trustworthiness, it is important to choose a suitable method of data collection. An interview-based method was chosen for this study in order to explore the experiences of health care staff in nursing for malnourished patients. The trustworthiness of this study was strengthened by the fact that three of the authors (AK, AW, VB) independently analyzed the text, then compared their analyses and agreed a common interpretation [27,31]. In order to facilitate the transferability of the results, it is important to give a clear description of the study context, the selection of respondents, the data collection and the analysis process [22,27]. Elucidating quotations were presented in the Results section in order to facilitate and confirm the trustworthiness of the interpretations [27].

To provide a comprehensive picture of the variation and width of the investigated in-hospital malnutrition, it must be viewed from several different perspectives. Because both expatriate and non-expatriate nurses assume primary responsibility for patient care in a ward, representative samples from both groups of nurses were selected to participate in the study. This increased the credibility of the results [27]. Presumably, a larger number of informants and an increasingly diverse selection could contribute to greater variety in the results. Both women and men need to give their opinion on the healthcare provided to diverse patient groups, and both sexes participated in this study. This means that the sample in this study is well representative of the staff compositions of other Saudi hospitals.

Polit et al. [15] argue that there are no rules for the sample size in qualitative research and that this is guided instead by the purpose of the study, the quality of the collected data, and the selection strategy. An interview guide was used in order to ensure that the interviews would ask questions relevant to this study and as a means for the interviewer(s) to get the informants to stay on topic. This yielded a rich material on which the analysis was based and enhanced the credibility of the results [27]. The last three interviews yielded no new information, but they confirmed the perceptions and experiences of the nurses as revealed in the earlier interviews and this strengthened the credibility of the results [27].

The Cultural Care Diversity and Universality theory has been selected as a framework to support analysis of the cultural aspects that emerged during the interviews in a multicultural environment and with nurses of diverse origins [18,32]. The Saudi Arabian health care system relies on expatriate physicians and nurses who comprise more than 80% of its total work force [15]. Health care staff from diverse cultures bring a variety of experiences to the practice settings [16,17]. The central purpose of the theory of Cultural Care Diversity and Universality is to “discover and explain diverse and universal culturally based care factors influencing the health, well-being, illness, or death of individuals or groups. The *purpose* and *goal* of the theory is to use research findings to provide culturally congruent, safe, and meaningful care to clients of diverse or similar cultures” [32].

Viewing the central themes of the present study (Potentials for nurses to provide good nutrition and physical activity, and Having the ability but not the power to provide good nutrition and physical activity for patients) in light of the Theory of Cultural Care Diversity and Universality, we can highlight culturally meaningful recommendations for care where culturally congruent care means providing care that is meaningful and fits with cultural beliefs and lifeways [18].

In this study it was shown that nurses suffered from a lack of dialog, where dialog could refer to both language channels and cultural communication. There might be several reasons behind the development of miscommunication and misunderstanding between nurses and patients. Some of these reasons can possibly relate to cultural factors and others to language barriers. Earlier studies such as Tate et al. [33] maintain that speaking different languages creates confusion and misunderstanding, while from the nurses’ side being aware of the patients’ cultural aspects will give the nurse an opportunity to provide holistic care. The cultural theorist Leininger [19] states that obtaining knowledge about patients’ social structure, factors such as religion, politics, economics, cultural history, life span values, kinship, and philosophy of life, helps the nurse to provide congruent care that can maximize wellness, prevent illnesses, alleviate cultural stresses, and help to sustain quality of cultural life [19]. Thus, it would be important to further educate expatriate nurses about the Saudi Arabian context, culture, religion, and language.

Furthermore, in our study, the presence of relatives during the patients’ hospital stay was experienced both as a resource and as limiting nurses’ opportunities to provide proper health care for the patients, especially with regard to their nutrition. The challenge of having relatives present during patients’ hospital stay in KSA has been described in previous studies [15]. The relatives or significant others can often influence the care that is provided and provide care themselves. Especially on female wards, a *Mourafiq* (relative or outside sitter) is often present during the hospital stay [15]. Saudi Arabian researchers like Aboul-Enein [17] say that it is important for expatriate nurses to recognize the significance of strong extended family ties among the majority of the Saudi Arabian population [17]. The challenge, of having a relative present during the whole of a patient’s hospital stay, is not common in western countries. Thus, from a medical perspective it seems highly important to inform the relatives in KSA about the specific nutritional needs the patients have in order to make them part of optimizing nutritional care. This recommendation is in line with the theory of Cultural Care Diversity and Universality [32], which recommends that health care staff obtain knowledge about every individual patient’s cultural background.

Aside from language barriers, which did not occupy any great space in the planning and execution of care plans for malnourished patients, the lack of cooperation, and the role of relatives are possible factors explaining poor nutritional care. For instance, when nursing care is not enough there is a need to involve other professionals such as dieticians [34]. In a previous study in KSA [7] it was shown that patients at risk of undernutrition did not receive oral supplements, energy-enriched meals or even consultation with a dietician. Thus, with support from the previous study and this one, there are several arguments that highlight the need to implement nutritional policies to facilitate good nutritional nursing care. In addition, the “denial” of the problem by nurses suggested by the conviction that patients were admitted with existing problems of undernutrition, increases the risk of patients not receiving adequate care. This is not unique to Saudi Arabia, as other studies have highlighted similar problems. For instance, in a Swedish study [3] it was shown that nutritional problems were “taboo” among nurses. The findings of this study indicate that undernutrition was not always recognized and talked about, which perhaps explains why nurses thought the problem did not develop during the hospital stay and would report that nutritional problems existed prior to a patient’s hospital admission. Thus, in order to facilitate the identification of undernutrition and the monitoring of a patient’s nutritional status, it is necessary to implement systematic screening for undernourishment and follow-up of weight status.

Since Saudi Arabia, like the rest of the world, is becoming globally multicultural, transcultural nursing that is directed toward holistic and congruent health care is challenging nurses and other care givers to think broadly so that ethical and moral factors become clearly evident in working with patients from different cultures [18]. Health care professions therefore need knowledge of the complex social structure, world view, and cultural context of the people of Saudi Arabia in order to promote culturally congruent care [17,19]. Building on these facts, one can argue that in KSA and elsewhere, there might be a need to raise nurses’ awareness of the importance of transcultural nursing when caring for the patients’ nutrition, in order to promote health, healing, and well-being. Specific to the KSA setting, which was shown in this study, is the need for improvement of nurses’ knowledge in Arabic language in order to provide health education about nutrition and lifestyle issues to both patients and their relatives. A cost-effective way to prevent non-communicable diseases in KSA and elsewhere would be to better acknowledge nurses as mediators for change working against malnutrition and physical inactivity.

## Conclusion

The findings of this study showed that nurses felt that they have the capacity and passion to continue managing their job assignments but that the health care system must pay attention to several obstacles that impede the nurse’s role as a health care provider. Health care policy makers could facilitate and better acknowledge nurses as possible mediators for improved nutritional care. For example, better communication could be facilitated among health care providers on all levels. Furthermore, in order to provide holistic care, nurses and all health care personnel could be encouraged to further improve their cultural understanding, offer culturally sensitive care and put the patients in cultural perspective at the same time as seeing them as individual clients. Future research emphasizing the patients’ perspective of their nutritional care could possibly increase cultural understanding. Specific to the KSA setting that was shown in this study is the need for improvement of nurses’ knowledge of the Arabic language in order to better provide health education about nutrition and lifestyle issues to both patients and their relatives.

## Abbreviations

KSA: Kingdom of Saudi Arabia.

## Competing interests

The authors declare that they have no competing interests.

## Authors’ contributions

AK, AW and VB conceived the study and independently conducted the qualitative analysis. AK had the main responsibility for performing the data collection and drafting the manuscript. Both ÖE, and HMA-H participated in the drafting the manuscript and revising it critically. All authors read and approved the final manuscript.

## Pre-publication history

The pre-publication history for this paper can be accessed here:

http://www.biomedcentral.com/1472-6955/13/29/prepub
